# Emerging Influenza Strains in the Last Two Decades: A Threat of a New Pandemic?

**DOI:** 10.3390/vaccines3010172

**Published:** 2015-03-18

**Authors:** Claudia Trombetta, Simona Piccirella, Daniele Perini, Otfried Kistner, Emanuele Montomoli

**Affiliations:** 1 Department of Molecular and Developmental Medicine, University of Siena, Via Aldo Moro, 53100 Siena, Italy; E-Mail: trombetta@unisi.it; 2 VisMederi Srl, Enterprise of Service in Life Sciences, Via Fiorentina 1, 53100 Siena, Italy; E-Mails: piccirella@vismederi.com (S.P.); perini@vismederi.com (D.P.); 3 Independent Consultant, Vienna 1221, Austria; E-Mail: otfried.kistner@chello.at or kistner@vismederi.com

**Keywords:** pandemic, novel influenza viruses, H7N9, H9N2, H10N8

## Abstract

In the last 20 years, novel non-seasonal influenza viruses have emerged, most of which have originated from birds. Despite their apparent inability to cause pandemics, with the exception of H1N1 swine influenza virus, these viruses still constitute a constant threat to public health. While general concern has decreased after the peak of the H5N1 virus, in recent years several novel reassorted influenza viruses (e.g., H7N9, H9N2, H10N8) have jumped the host-species barrier and are under surveillance by the scientific community and public health systems. It is still unclear whether these viruses can actually cause pandemics or just isolated episodes. The purpose of this review is to provide an overview of old and novel potential pandemic strains of recent decades.

## 1. Introduction

A pandemic is defined by the Dictionary of Epidemiology as “an epidemic occurring worldwide, or over a very wide area, crossing international boundaries, and usually affecting a large number of people” [1]. The conditions necessary for a pandemic event are the outbreak of a novel influenza virus, its ability to cause illness in humans and its capacity to transmit from human to human [2].

A novel influenza virus is a novel subtype of haemagglutinin (and neuraminidase) to which humans have very little or no immunity. It could be the consequence of reassortment among seasonal human viruses and a virus originating in animals, mainly birds. Indeed, bird viruses display all 16 subtypes of haemagglutinin, while only three subtypes (H1, H2 and H3) are found in human influenza viruses [3].

The first pandemic event of the 21st century was caused by the new influenza strain H1N1, which appeared in Mexico in February and March 2009 [4]. Of swine origin, this virus was an example of triple reassortment and contained gene segments never previously reported in swine or human influenza viruses [5,6]. It has been estimated that, during the first year of the H1N1 pandemic, deaths worldwide ranged from 151,700 to 575,400, 80% of which involved persons younger than 65 years old, who are usually the main target of seasonal influenza epidemics [7]. In this regard, studies have shown pre-existing cross-protection against the H1N1 influenza virus in persons older than 60 years [8,9], which could explain why H1N1 influenza spread chiefly among younger subjects. Indeed, in a mouse model, Skountzou *et al.* [10] demonstrated that both pre-1957 influenza strains and old H1N1 strains induce cross-protection against the H1N1 influenza virus. 

Although H1N1 displayed the features of a pandemic strain, such as the ability to transmit from human to human and to cause illness [6], it was, nevertheless, relatively mild [11]. Possible explanations for this could be pre-existing immunity, the lesser capability of the virus to bind to and replicate in the target cells, or a decreased secretion of molecules that could cause further complications [11].

By contrast, the H5N1 virus, while potentially much more dangerous than swine H1N1, has not yet caused a pandemic. The reason is that the avian H5N1 virus has never been able to transmit efficiently from human to human [2], which, as mentioned above, is one of the main features of a novel potentially pandemic strain. Nevertheless, the H5N1 virus did prove able to transmit from poultry to humans [12]. Initially identified in Hong Kong in 1997, the virus spread worldwide, infecting 649 people in 16 countries and displaying a mortality rate of 60% [13]. This high mortality rate was presumably due to the fact that most humans do not have immunity [2] and to the strain’s specificity for α2,3 receptors, which are mainly present in the lower respiratory tract. 

The H5N1 virus remains a concern because it can mutate rapidly and acquire genes from viruses able to infect other animal species. Moreover, humans could be infected simultaneously by human and avian influenza viruses; these “mixing vessels” could result in a novel influenza subtype with sufficient human genes to allow easier human to human transmission [14].

In addition to H5N1, other avian viruses with a capacity for reassortment have been identified. In May 2014, the World Health Organization (WHO) reported the first human case of infection by the H5N6 influenza virus in China [15], while in May 2013 the first case of human infection by H6N1, a novel avian influenza virus, was identified in Taiwan [16].

Other emerging avian viruses belong to the H7 subtype. While H7N9 could give rise to the greatest concern, other viruses, such as H7N3 in 2004 [17], H7N2 [18] and H7N7 in 2003 [19], have been identified in humans. Public concern was also aroused by other novel influenza strains emerging in 1999 (H9N2) and 2013 (H10N8).

Vaccines and antiviral drugs are the only effective ways to prevent influenza infection and treat illness [20], and their availability plays a key role in the event of a pandemic. During the relatively mild 2009 H1N1 influenza pandemic, vaccines did not become available until six months after the pandemic strain had been identified [21]; as a result, more than 90% of the world’s population did not have timely access to vaccines and antiviral drugs [22].

One way to tackle pandemics could be to implement a “split strategy”. This consists of priming the naïve population with a single dose of a representative pre-pandemic vaccine, such as H5 or H7, and following this up with the administration of a vaccine containing the emerging strain. The advantages of this strategy are two-fold. Firstly, only one dose is initially administered; secondly, the subsequent dose can be administered promptly in order to better match the emerging virus [23,24].

In a pandemic situation, live attenuated vaccines (LAIV) appear to be more appropriate than inactivated vaccines, as they are able to induce humoral and cellular immune responses locally at the initial site of infection and to mimic the immune response after natural infection [3,25]. LAIV vaccines are more effective in young children and adolescents [3,26] and more protective not only against well-matched viruses but also in the case of antigenic drift from the vaccine antigen [27]. 

The disadvantage of LAIV vaccines is the risk of transmission and reassortment of the virus, which could lead to the emergence of increased virulent viruses [28,29,30]. Such a risk could be reduced conducting clinical trials in an inpatient isolation setting when influenza viruses do not likely circulate [31,32], limiting the deployment of LAIV vaccines until after the emergence of pandemic, comprehending the presumed shedding patterns and determining the biological behavior of potential reassortment between vaccine and wild type virus [29,30]. Seasonal and pandemic LAIV vaccines have been approved and licensed for use in the USA and Russia [26,33]. Studies have shown low shedding in pandemic LAIV viruses [32] and the lack of virus transmission from vaccinees to unvaccinated persons [31,34,35].

By contrast, split and subunit vaccines are poorly immunogenic; they therefore need high doses and more administrations in order to elicit a significant immune response [26,36]. Adjuvants may help to improve the immunogenicity of conventional split and subunit vaccine preparations, as adjuvanted vaccines induce a broader immune response than nonadjuvanted vaccines. Moreover, as adjuvanted vaccines use a smaller amount of antigen, a higher number of doses can be produced [3,26].

The purpose of this review is to provide an overview of the novel potential pandemic influenza strains that have appeared in the last two decades, while focusing particularly on H7N9, H9N2 and H10N8.

## 2. Emerging Influenza Strains

### 2.1. H7N9 Influenza Virus

The first three cases of infection with a novel influenza A H7N9 virus were reported by the Chinese Center for Disease Control and Prevention in February and March 2013 [37]. The patients were two men aged 87 and 27 years and a woman aged 35 years. All three patients had underlying medical condition and died [38]. By the end of May 2014, a total of 439 cases of H7N9 infection had been reported to the WHO [39], with a case fatality rate of 30% [40].

The virus rapidly spread out of 14 provinces in China [41] and in April 2014 the first case outside China was reported [42]. Two waves were identified, the first from February to May 2013, with 133 cases, and the second from October 2013 to February 2014 [40]. While cases occurred in both men and women over a wide age-range [40], 56% of cases involved subjects aged over 60 years [43], mostly males [40]. This age-related pattern could be explained by the fact that the infection was mild or asymptomatic in children and may have gone undiagnosed. Moreover, poultry markets are mostly frequented by adults, and some patients were poultry workers. In addition, as this was the first report of H7N9 infection, the population was presumably naïve to the novel virus, which displayed a propensity to infect vulnerable people, such as the elderly or those with underlying complications or impaired immunity. The reason why males were mainly infected could be that poultry markets were frequented by more men than women [39,42,43,44].

Live poultry markets were identified as the main source of human infections, since most of the infected patients had been exposed to poultry. However, the novel H7N9 virus was asymptomatic in poultry, which made it difficult to detect infected farms and to identify the source and mode of transmission [39,45,46].

The novel H7N9 virus was of avian origin and phylogenetic analysis showed that it was the result of triple reassortment. The HA gene originated from the duck H7N3 subtype, the NA gene from a migratory bird H7N9 virus in Korea, and the six internal genes from poultry H9N2 viruses [6,38,46,47]. Domestic ducks are the intermediate host of H7N9, which is reassorted with H9N2 when transmitted to chickens [48]. Its adaptation and transmission to domestic poultry may be connected to the deletion of five amino acids in the NA stalk, a feature that has also been detected in the H5N1 avian virus [38].The low pathogenicity of H7N9 in poultry is due to the HA cleavage site, which has only a single basic amino acid (arginine), while its severe pathogenicity in humans might be associated with the internal genes from H9N2 [38,44].

It seems that H7N9 can be transmitted from poultry to humans more easily than H5N1, given that 77% of H7N9-infected patients had previously been exposed to poultry [46], while human-to-human transmission proved inefficient and difficult. Some studies have indicated a possible transmission of the novel H7N9 virus among family members; indeed, genetic factors may facilitate transmission of the virus, and potential genetic susceptibility could play an important role. Although human-to-human transmission of the H7N9 virus is limited, the fact that the virus undergoes continuous reassortment should not be underestimated [49,50,51].

Common serological assays used to quantify influenza antibodies are Hemagglutination Inhibition (HI) and Virus Neutralization (VN). However, the HI assay appears to be insensitive or not standardized for avian influenza viruses, as it detects no or low HI titers [52,53]. The WHO suggested a modified HI protocol in order to improve its sensitivity to H7N9 virus [54]. It could be crucial to understand the immune response of H7 vaccines in case they elicit an immune response directed against other regions of the HA gene without inducing an HI antibody response [54,55,56,57]. Indeed, this phenomenon has been described in studies in which people with documents infection do not have serological antibody response [19,58] or in which the vaccine has proved to be protective in animal models even if no or low HI titers are found [59].

A recent study by Wang *et al.* [60] showed that a relatively high proportion of poultry workers have been exposed to H7. This finding suggests that mild or asymptomatic infections by H7N9 are recurrent in this high-risk population subset. In the case of seroprevalence studies, too, the technique employed may heavily influence results by either overestimating or underestimating antibody titers. 

No H7N9 vaccines are currently available, although the WHO has recommended using an A/Anhui/1/2013-like virus for their development [61]. Various platforms have been evaluated in order to create efficacious influenza H7N9 vaccines. Chen *et al.* [62] demonstrated the high immunogenicity and efficacy of a live attenuated H7N9 vaccine candidate obtained by reverse genetics in a ferret challenge. Their vaccine displayed high yield in eggs, conferred complete protection after one dose against the homologous virus and cross-reacted to the H7N7 virus. It is currently being evaluated in phase-I clinical studies. Other studies [63,64,65] have evaluated the efficacy in mice of inactivated whole virus vaccines, either as wildtype or reverse genetics reassortant and of a whole virus particle vaccine in ferrets. These vaccines proved able to attenuate the impact of the disease and reduce both the severity of infection and viral load.

Adjuvanted split and subunit vaccines may also constitute an efficacious means of combating pandemic disease, owing to the ability of adjuvants to improve the immune response and reduce the amount of antigen needed [66,67]. Studies [59,68] have shown the benefit of squalene-based and aluminum-salt adjuvants, which are able to enhance the immune response after two doses and to improve vaccine efficacy. An oil-in-water emulsion, MF59, is an adjuvant able to induce a greater antibody response than other adjuvants [66]. A phase-I clinical study has demonstrated the efficacy of a cell culture-derived MF59-adjuvanted H7N9 vaccine, which has proved to be immunogenic in naïve subjects after two doses [66]. Other study compared the immune response to A/Shanghai/2/13 (H7N9) influenza vaccine with or without the MF59 adjuvant. The results showed that 59% of participants reached seroconversion after two lowest doses of antigen vaccine with MF59 adjuvant while without adjuvant the minimum antibody response has been reached by the highest antigen dosage [69]. The previous experience with H5N1 and H1N1 pandemic split vaccines shows the advantage of the MF59 adjuvant able to induce good immune response in a broad population with a low formulation dosage [70,71,72].

The production process of influenza vaccines is time-consuming and, in the event of a pandemic, an initial strategy could be to use the existing H7 live-attenuated vaccines, which are able to induce a protective antibody response [67]. Indeed, it has been shown that H7N3live attenuated and H7N1 vaccines are able to induce cross-reactivity to the H7N9 virus [56,67,73].

The appearance of a novel H7N9 virus raises global public concerns because this virus had never been detected in humans or animals. Consequently, all age-groups worldwide might be susceptible. In addition, analysis of its gene sequences has shown that the virus has great potential to infect mammals [74]. The emergence of the novel H7N9 virus shows that humanity must always be alert for the next pandemic and that virological surveillance of wild birds and domestic poultry is an important means of understanding the emergence of novel avian influenza viruses in future [42].

### 2.2. H9N2 Influenza Virus

The first cases of infection with an H9N2 influenza strain were reported in China and affected two children. Patient 1 suffered mainly from fever, sore throat, headache, abdominal pain, vomiting, inflamed oropharynx and lymphopenia. The second patient was admitted to hospital with fever, vomiting, poor appetite and an inflamed oropharynx [75]. Subsequently, serological analysis conducted on samples from human subjects in Southern and Northern China found that about 1% of the subjects tested had antibodies against the H9 subtype hemagglutinin [76,77]. From a phylogenetic point of view, the H9N2 strains can be grouped into three main sub-lineages: A/Qa/HK/G1/97 (G1-like), A/Dk/HK/Y280/97 (Y280-like) and A/Ck/Korea/38349-p96323/96 (Korean-like) [78,79,80]. The symptoms of infection with H9N2 are mild in comparison with those of H5N1 infection. Of particular interest, however, is the fact that the internal genes of H9N2 are similar to those of the H5N1 that caused the infections in 1997. Indeed, Guan *et al.* [79] concluded that H5N1 strains originated as a re-assortment of the QayHKyG1y97-like strain on the basis of their closely related internal genes. For this reason, H9N2 was regarded as a potential pandemic strain, or at least as a candidate for a mock-up influenza vaccine. H9N2 influenza viruses have human virus-like receptor specificity [81] and potentially they could cross the species barrier more efficiently than H5N1 strains [78]. The first Vero cell–derived non adjuvanted whole virus H9N2 vaccine was therefore developed [82] and subsequently successfully tested in a clinical study, which demonstrated its excellent safety profile and high immunogenicity [83].

### 2.3. H10N8 Influenza Virus

A novel avian influenza strain, H10N8, showed up in humans by the end of 2013. So far, three cases have been recorded, all in China, with two fatal outcomes [84]. The first and most well documented case of H10N8 was detected in the Jiangxi region. The patient, a 73 years old woman with a clinical history of chronic diseases, was admitted to hospital on 30 November 2013 and died a week later. A subsequent study [85] showed that a poultry market, which had been visited by the patient a few days before her hospitalization, was the most probable cause of infection.

H10N8 was first isolated in Italy in wild birds [86] and was subsequently found in various countries, including China. Gene sequencing of the virus isolated from the first patient revealed that all internal genes had originated from an H9N2 strain. This point is of particular interest, since it has been shown that H9N2 also donated internal genes to the H5N1 and H7N9 virus strains that have caused occasional infections of humans in recent years [38,79]; it could therefore constitute a particularly effective viral genetic “reservoir” in human infection. Qi *et al.* [85] showed that H10N8 had undergone many re-assorting events; this conclusion is supported by the fact that, unlike the commonly highly pathogenic H5N1 and lowly pathogenic H7N9, it causes variable pathogenicity in wild and domestic birds. This high genetic variance has given rise to adaptation for mammalian infection, which was observed in wild dogs in the vicinity of poultry market where the first human patient was infected [87].

It is still very hard to understand the actual threat posed by H10N8 in terms of a possible pandemic event. Despite the fact that it apparently displays high mortality, too few cases have been reported to set any reliable statistics. Moreover, no further cases of human infection have been reported since February 2014, and human-to-human transmission, as for the other recent avian influenza viruses, appears to be difficult or impossible. It should be taken into account, however, that H10N8 has shown a particularly high capacity for gene re-assortment in birds, which could give rise to dangerous adaptive mutations. Notably, the neuraminidase of subtype N8 is not a first-timer, since it has been shown to be the one of the neuraminidase constituents of one of the first recorded pandemics in human history (“Russian” flu of 1889–1890) [88].

## 3. Conclusions

Influenza viruses undergo genetic variations resulting in diverse novel strains to which humans have little or no immunity [55,89]. The range of hosts is very broad, including humans, pigs, horses and sea mammals. Combination of one of the hemagglutinin subtypes with one of the neuraminidase subtypes gives rise to wide genetic diversity and could lead to a pandemic event [89].

Avian influenza viruses are able to spread from the natural reservoir to domestic poultry and subsequently to humans. As pigs have both human (SA α2,6-Gal) and avian receptors (SA α2,3-Gal), they constitute “mixing vessels” in which viruses can reassort, mutate, adapt and may acquire the ability to infect humans [55].

In the last two decades, and especially in the last two years, a number of novel avian influenza strains, such as H7N9, H10N8 and H6N1 in 2013 and H5N6 in 2014, have emerged. The appearance of novel reassorted viruses able to infect humans is cause for concern because it increases the probability that a strain will acquire human-to-human transmission capability, making the emergence of an influenza pandemic more likely [90].

Until now, these novel influenza strains have developed a very low or non-existent capacity for human-to-human transmission. Nevertheless, the possibility of further adaptation of the virus in humans and the acquired capability of sustainable human-to-human transmission must be considered.

The human H9N2 viruses possess a set of internal genes found also in H5N1, H7N9, and H10N8. It could be interesting to investigate whether and/or how this particular set of genes can facilitate the jump from poultry to humans and cause disease [91]. 

Furthermore, the number of human cases of infection by novel influenza viruses may have been underestimated by the techniques used in surveys. Considering that RT-PCR and viral culture are able to detect only active infection, the possibility of using other serological techniques should be considered, in order to ascertain the true prevalence of the disease, to identify mild or asymptomatic infections and to detect other subtypes or avian viruses that could infect humans [92]. Many serological techniques, however, need to be optimized, as they sometimes underestimate antibody titers against avian viruses.

Serological surveillance in humans and animals, such as wild birds and poultry, remains the key to controlling and understanding the emergence of novel avian influenza strains. Mankind needs to be prepared for the next possible pandemic despite, or even because of, the difficulty of predicting which strain may cause the next pandemic.
